# Interactive Effects of Blue Light and Water Turbulence on the Growth of the Green Macroalga *Ulva australis* (Chlorophyta)

**DOI:** 10.3390/plants13020266

**Published:** 2024-01-17

**Authors:** Hojun Lee, Stephen Depuydt, Kisik Shin, Jonas De Saeger, Taejun Han, Jihae Park

**Affiliations:** 1 Bio Environmental Science and Technology (BEST) Lab, Ghent University Global Campus, 119-5, Songdomunhwa-ro, Incheon 21985, Republic of Korea; 2 Erasmus Brussels University of Applied Sciences and Arts, Nijverheidskaai 170, 1070 Brussels, Belgium; 3 Water Environmental Engineering Research Division, National Institute of Environmental Research (NIER), 42, Hwangyeong-ro, Incheon 22689, Republic of Korea; 4 Department of Animal Sciences and Aquatic Ecology, Ghent University, Coupure Links 653-Block F, B-9000 Gent, Belgium; 5 Center for Environmental and Energy Research, Ghent University Global Campus, 119-5, Songdomunhwa-ro, Incheon 21985, Republic of Korea

**Keywords:** aquaculture, blue light, growth, macroalga, *Ulva australis*, water turbulence

## Abstract

Macroalgal growth and yield are key to sustainable aquaculture. Although light and water turbulence are two important factors that affect algal productivity, research on their interaction is limited. Therefore, in this study, we investigated the effects of different wavelengths of light and the presence or absence of water turbulence on the growth of the green macroalga *Ulva australis*. Water turbulence was found to enhance the growth of *U. australis* irrespective of photosynthetic performance, but only in blue light cultures. The quantum dose of blue light required to induce 50% growth promotion was 1.02 mol m^−2^, which is comparable to the reported values for cryptochrome-mediated effects in other macroalgae. The combined effect of blue light and water turbulence led to the accumulation of photosynthesis-related proteins that support plastid differentiation and facilitate efficient photosynthesis and growth. Our findings thus highlight the potential of harnessing blue light and water turbulence to maximise macroalgal cultivation for sustainable and profitable algal aquaculture.

## 1. Introduction

Marine macroalgae or seaweeds have diverse uses, including in food, animal feed, fertilisers, and as raw materials for cosmetics and biofuels [1,2]. Aquaculture of seaweeds is an emerging industry that offers a sustainable solution to alleviate food scarcity as well as related environmental issues [1,2,3]. Consequently, identifying the optimal growth conditions for seaweed is critical to increasing yields and promoting the sustainable production of seaweed-based products across multiple industries [2,4].

Seaweed growth and productivity are influenced by environmental conditions, including light, nutrient availability, salinity, and temperature. Of these, light is of paramount importance because it plays a pivotal role as an energy source and information carrier. Non-optimal illumination conditions, such as insufficient or excessive light, can adversely affect the ecology and profits of seaweed farms [5,6]. Moreover, the spectral composition of light regulates various aspects of algal biology, such as the production of photosynthetic pigments, gene expression mediated by photoreceptor signalling pathways, and developmental changes in the algal lifecycle [7,8,9].

The crucial role of blue light in marine macroalgal morphogenesis and photosynthesis is well documented [10]. Blue light influences algal metabolism and development, thereby promoting photosynthetic capacity, which improves algal survival and facilitates spore germination in brown algae [11,12,13]. In *Saccharina*, *Laminaria*, and *Undaria*, blue light stimulates growth and affects gametophyte production [8,14,15,16,17]. Furthermore, blue light facilitates the recovery of UV-B-irradiated spores and thallus disks of *Ulva australis*; additionally, it stimulates germination and photosynthesis [18]. According to these findings, the growth of *Ulva lactuca* and *U. australis* may be stimulated by exposure to blue light [7,18]. Investigating the effects of blue light on the growth and development of marine macroalgae, such as *U. australis*, has thus contributed to the research on optimising seaweed aquaculture practices [9].

Water turbulence is another crucial factor affecting seaweed growth and development [19,20,21,22]. It plays a vital role in the transport of inorganic carbon and nutrients essential for algal survival and growth [23]. Turbulence enhances nutrient uptake and growth by reducing the diffusion boundary layer along the algal surface [24,25]. In comparison to low-turbulence water environments, moderate water turbulence promotes the growth and productivity of macrophytes [24,26]. During turbulent conditions, the growth rate of various kelp species was found to depend on the trade-off between tissue strength and blade growth rate [27,28,29]. Furthermore, the sensitivity of different seaweed species to water turbulence affects their development in different ways [30]. For instance, *Laminaria digitata* exhibited a reduced thallus growth rate at both low and high water velocities, whereas *Laminaria hyperborea* was not directly affected by turbulence [31].

However, the effect of water turbulence on growth is influenced by other factors, such as light intensity and nutrient concentration [32,33,34,35]. Although the effects of water turbulence on the growth of commercially important algal species have been extensively investigated, the results have been inconsistent. This could be attributed to the effects of water turbulence being obscured or influenced by additional environmental factors. Therefore, understanding the effects of water turbulence on algae and optimising water velocity in algal aquaculture settings can improve the growth and productivity of target algal species and, consequently, the seaweed farming industry’s sustainability.

Among many seaweeds, several characteristics of *Ulva* spp., such as rapid growth rate, high productivity, and nutrient-rich composition, render it an excellent candidate for aquaculture [36]. Furthermore, it is well known that *Ulva* spp. can function as effective bioremediation agents by absorbing excess nutrients and pollutants from wastewater and coastal areas, thus contributing to the restoration of water quality [37].

*U. australis*, previously known as *Ulva pertusa*, is an ecologically and economically significant widely distributed macroalga. This macroalga forms ‘green tides’ in coastal areas of Asia and the Mediterranean and its sensitivity to pollutants makes it a model species for water quality assessment [18,38,39,40,41,42,43,44]. *U. australis* is also a valuable food source in many Asian countries [45] and exhibits algicidal and therapeutic properties attributed to its bioactive compounds [46,47,48]. Thus, investigating the factors that influence the growth of *U. australis* may be important for its sustainable development and the conservation of coastal ecosystems. Although the effects of blue light on *U. australis* growth have been studied [18], the individual effects of water turbulence and the combined effects of blue light and water turbulence on this species remain unexplored. Therefore, understanding the effects of blue light and water turbulence on the growth and development of *U. australis* can unlock its full potential in the food, feed, and biotechnology industries, among others, while minimising its ecological footprint.

This study aimed to examine the effects of water turbulence and various wavelengths of light on the growth and physiology of *U. australis*. Accordingly, *U. australis* was cultivated under white, blue, green, or red light conditions, both with and without water turbulence (aeration) and its growth, photosynthetic capacity, and biochemical composition were evaluated for each treatment. Furthermore, growth was modelled as a function of the quantum dose to determine the light intensity required for eliciting a 50% growth response. Proteins that accumulated under growth-promoting conditions were identified.

## 2. Results

### 2.1. Growth and Cell Number

**Figure 1:**
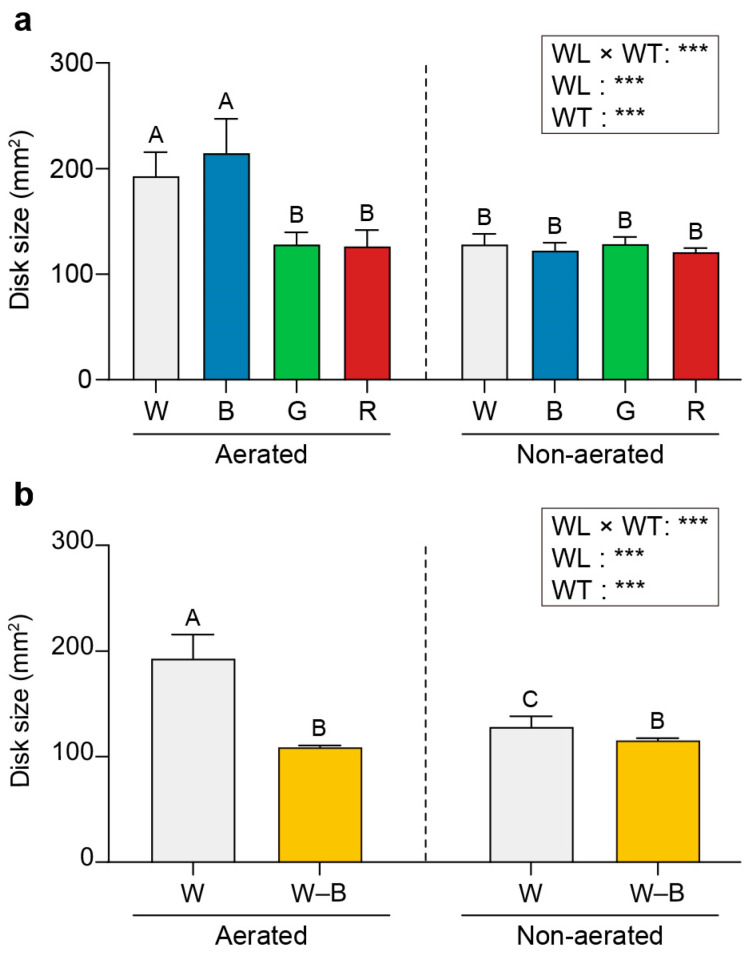
Effects of wavelength and water turbulence on *Ulva australis* growth. Disk size (mm^2^) under white, blue, green, or red light (**a**) and white light or white light depleted of blue wavelengths by filtering (**b**). Data are presented as the mean ± 95% confidence intervals (*n* = 3, at least five *Ulva austaralis* plants per replicate). Different letters indicate differences at *p* < 0.05. W, white light; B, blue light; G, green light; R, red light; W–B, white light with blue wavelength filtered out; WL, wavelength; WT, water turbulence; *** *p* < 0.001.

Disks collected from *U. australis* thalli were cultured under white, blue, green, or red light illumination, with or without water turbulence generated by aeration. Significantly higher growth rates were observed under turbulent conditions illuminated with white and blue light (193.16 and 214.85 mm^2^, respectively) than those illuminated with green and red light (128.42 and 126.49 mm^2^, respectively; Figure 1a). No statistically significant difference in growth rates was observed among the light treatments under static conditions.

The growth of *U. australis* was compared under white light with or without the blue wavelength, otherwise maintaining the same fluence rate. The growth of the disks cultured under white light devoid of the blue waveband was only half that of the disks cultured under white light (Figure 1b).

**Figure 2:**
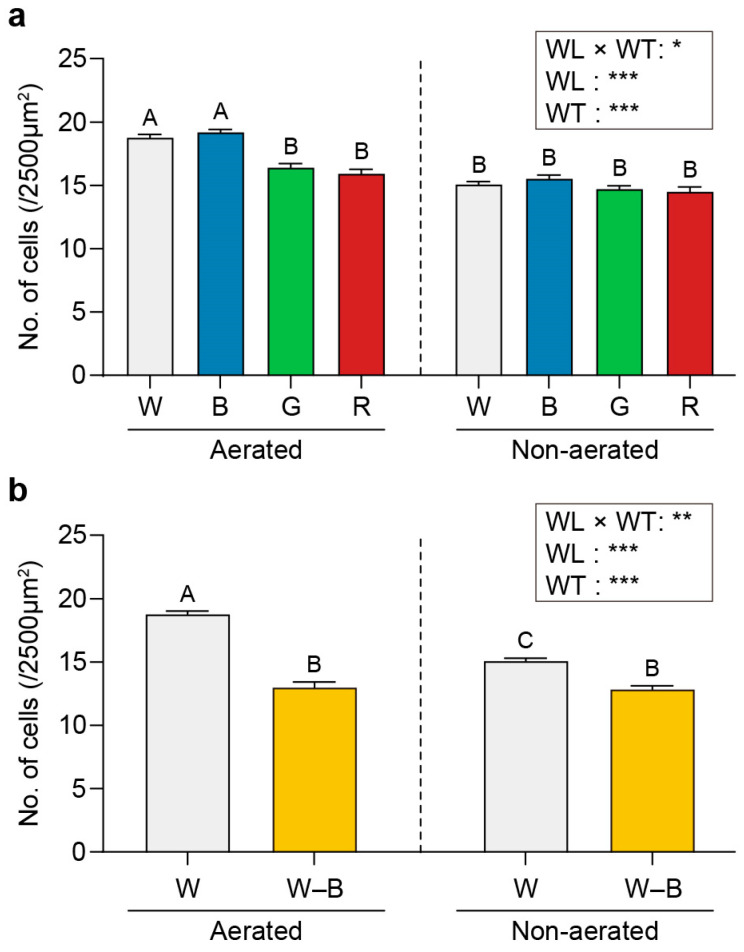
Density of *U. australis* cells per unit area as a function of exposure to different light wavelengths and water turbulence. White, blue, green, or red light (**a**) and white light or white light with blue wavelengths filtered out (**b**). Data represent mean ± 95% confidence intervals (*n* = 3, at least five *Ulva austaralis* plants per replicate). Different letters indicate differences at *p* < 0.05. W, white light; B, blue light; G, green light; R, red light; and W–B, white light with blue wavelength filtered out. WL, wavelength; WT, water turbulence; * *p* < 0.05; ** *p* < 0.01; and *** *p* < 0.001.

Under aerated conditions, *U. australis* cell density was significantly higher when grown under white and blue light (18.78 and 19.19 cells/2500 μm^2^, respectively) than when grown under other wavelengths (16.42 cells/2500 μm^2^ for green light and 15.94 cells/2500 μm^2^ for red light; Figure 2a).

However, no significant differences were observed in cell number among light wavelengths under static conditions (15.08, 15.53, 14.72, and 14.53 cells/2500 μm^2^ for white, blue, green, and red light, respectively; Figure 2a). *U. australis* cell density was lower in the absence of blue light (13 and 12.83 cells/2500 μm^2^ for aerated and static conditions, respectively; Figure 2b) than under full white light for both aerated and static conditions.

### 2.2. Photosynthetic Pigment Contents

**Figure 3:**
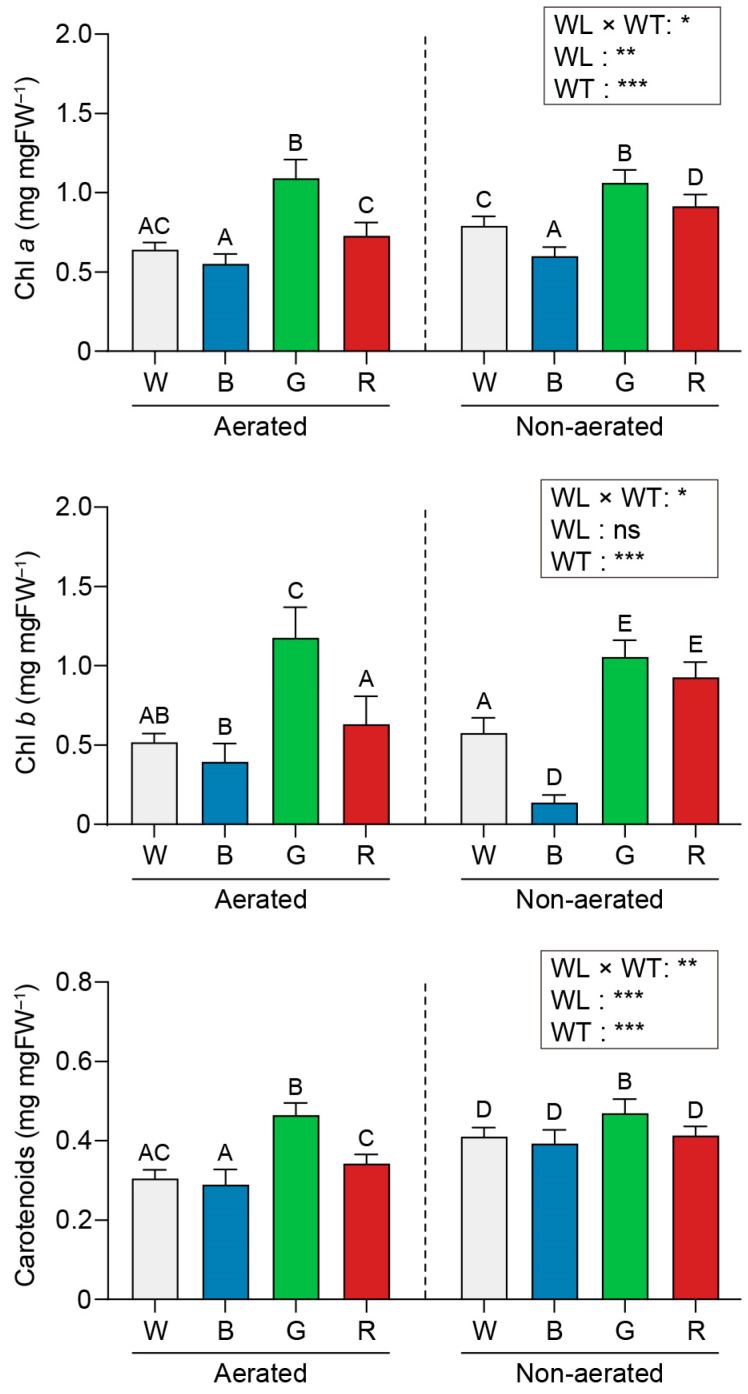
Effects of different light wavelengths and water turbulence on pigment contents. All data are expressed in mg per mg fresh weight (mg·mgFW^−1^). W, white light; B, blue light; G, green light; and R, red light. WL, wavelength; WT, water turbulence; ns, not significant; * *p* < 0.05; ** *p* < 0.01; and *** *p* < 0.001. Data represent mean ± 95% confidence intervals (*n* = 3, at least five *Ulva austaralis* plants per replicate). Different letters indicate differences at *p* < 0.05.

**Figure 4:**
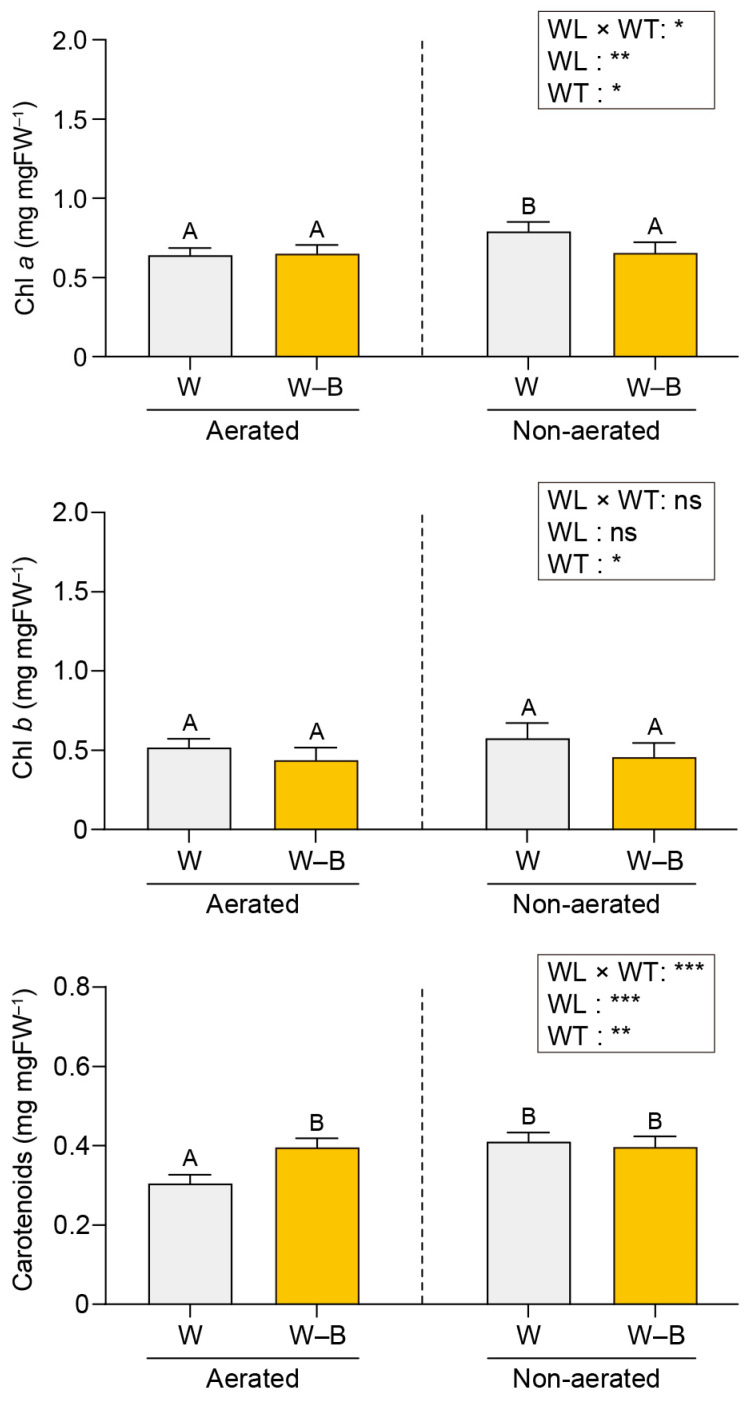
Effects of blue light and water turbulence on photosynthetic pigment concentrations in *U. australis*. All data are expressed in mg·mgFW^−1^. W, white light and W–B, white light with blue wavelength filtered out. WL, wavelength; WT, water turbulence; ns, not significant; * *p* < 0.05; ** *p* < 0.01; and *** *p* < 0.001. Data are presented as the mean ± 95% confidence intervals (*n* = 3, at least five *Ulva austaralis* plants per replicate). Different letters indicate differences at *p* < 0.05.

The photosynthetic pigment contents of *U. australis* under each experimental condition are shown in Figure 3 and Figure 4.

Regardless of aeration, green light yielded the highest pigment concentrations in *U. australis* disks compared with other lights (chlorophyll *a* (Chl *a*): 1.09 and 1.06 mg per mg fresh weight (mg·mgFW^−1^), Chl *b*: 1.18 and 1.06 mg·mgFW^−1^ in aerated and static cultures, respectively, and carotenoids: 0.47 mg·mgFW^−1^ in both aerated and static cultures). The lowest pigment concentrations were observed under blue light (Chl *a*: 0.55 and 0.60 mg·mgFW^−1^, Chl *b*: 0.40 and 0.14 mg·mgFW^−1^, and carotenoids: 0.29 and 0.39 mg·mgFW^−1^) in aerated and static cultures; Figure 3).

The photosynthetic pigment contents were comparable under full white light and white light devoid of the blue wavelength and in both the aerated and static culture conditions (Figure 4).

### 2.3. Chl a Fluorescence

**Figure 5:**
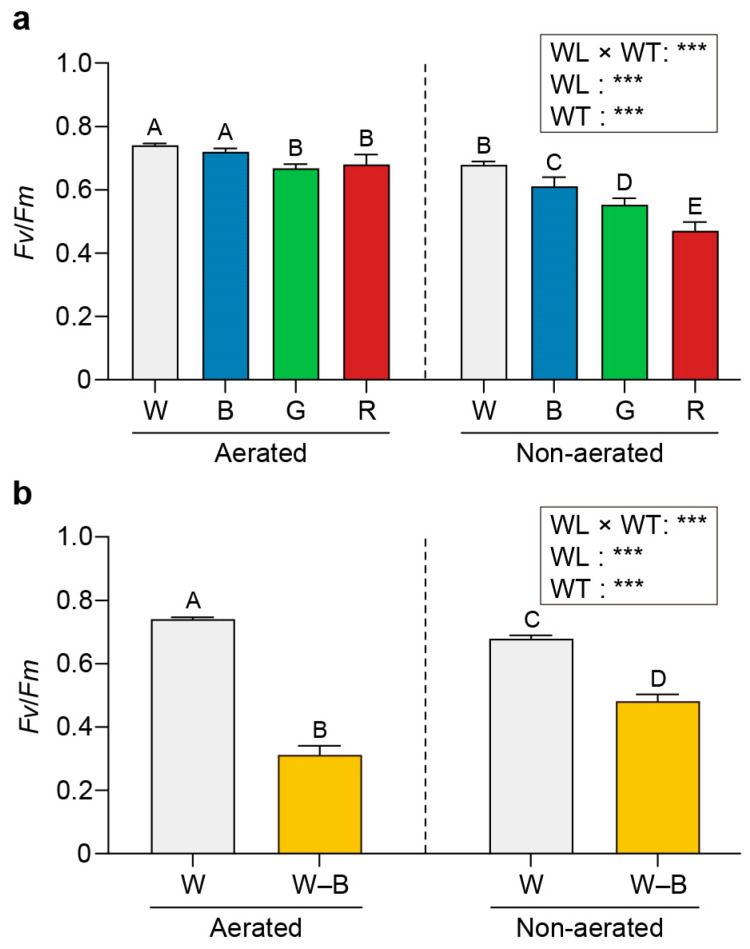
Effects of light exposure and water turbulence on the maximum quantum yield (*Fv/Fm*). *Fv/Fm* values for *U. australis* grown under white, blue, green, or red light (**a**) and white light or white light with blue wavelengths filtered out (**b**). Data are presented as the mean ± 95% confidence intervals (*n* = 3, at least five *Ulva austaralis* plants per replicate). Different letters indicate differences at *p* < 0.05. W, white light; B, blue light; G, green light; R, red light; and W–B, white light with blue wavelength filtered out. WL, wavelength; WT, water turbulence; *** *p* < 0.001.

**Figure 6:**
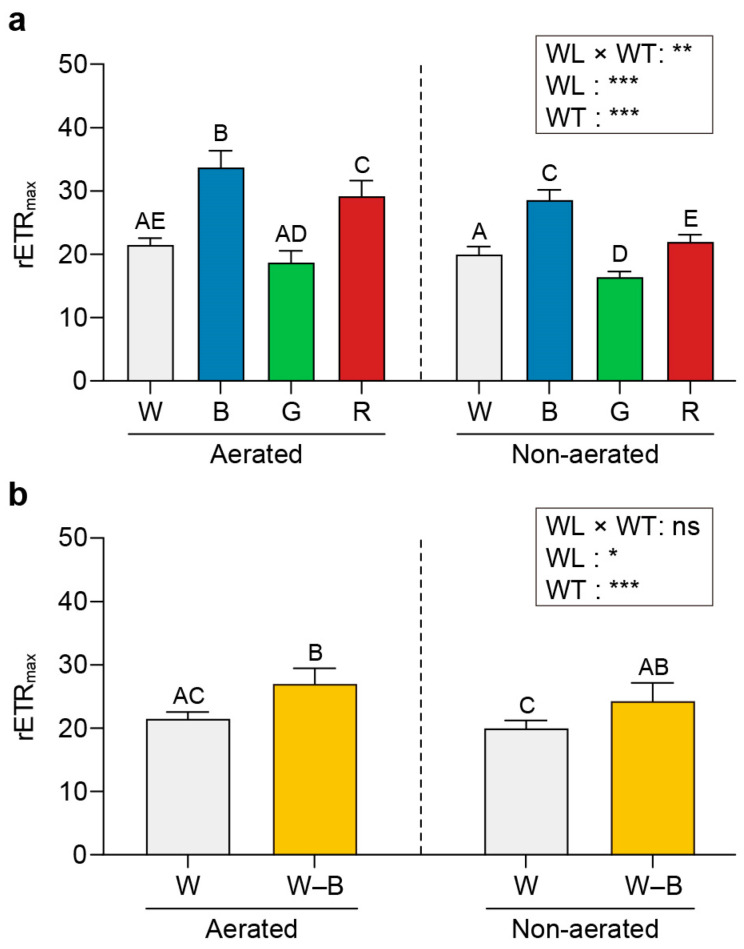
Effects of light and water turbulence on the relative maximum electron transport rate (rETR_max_). rETR_max_ values for *U. australis* grown under white, blue, green, or red light (**a**) and white light or white light with blue wavelengths filtered out (**b**). Data are presented as the mean ± 95% confidence intervals (*n* = 3, at least five *Ulva austaralis* plants per replicate). Different letters indicate differences at *p* < 0.05. W, white light; B, blue light; G, green light; R, red light; and W–B, white light with blue wavelength filtered out. WL, wavelength; WT, water turbulence; ns, not significant; * *p* < 0.05; ** *p* < 0.01; and *** *p* < 0.001.

**Figure 7:**
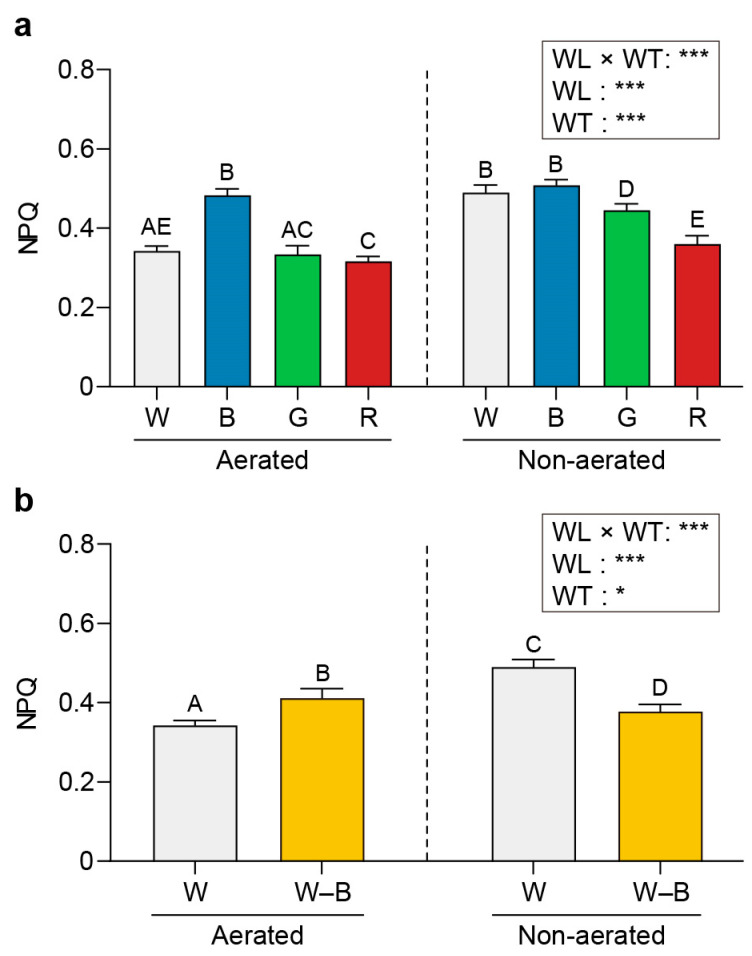
Effects of light and water turbulence on non-photochemical quenching (NPQ). NPQ values for *U. australis* grown under white, blue, green, or red light (**a**) and white light or white light with blue wavelengths filtered out (**b**). Data are presented as the mean ± 95% confidence intervals (*n* = 3, at least five *Ulva austaralis* plants per replicate). Different letters indicate differences at *p* < 0.05. W, white light; B, blue light; G, green light; R, red light; and W–B, white light with blue wavelength filtered out. WL, wavelength; WT, water turbulence; * *p* < 0.05; and *** *p* < 0.001.

Figure 5, Figure 6 and Figure 7 present the maximum quantum yield (*Fv/Fm*), relative maximum electron transport rate (rETR_max_), and the non-photochemical quenching (NPQ) values estimated for *U. australis* grown under different wavelengths of light and turbulence conditions.

The *Fv/Fm* values were higher upon exposure to white (0.74) and blue light (0.72) than upon exposure to green (0.67) or red light (0.68) under aerated conditions (Figure 5). Under static conditions, the *Fv/Fm* values varied significantly among the different wavelengths (0.68, 0.61, 0.55, and 0.47 for white, blue, green, and red light, respectively; *p* < 0.05).

The rETR_max_ was highest under blue light (33.75) and lowest under green light (18.74); however, it presented an intermediate value under red light (29.19; Figure 6). Blue light induced a higher NPQ value than that induced by other wavelengths under both aerated and static conditions (0.48 and 0.51, respectively; Figure 7).

### 2.4. Correlation between Growth, Photosynthetic Efficiency, and Photosynthetic Pigment Content of U. australis under Different Light Wavelengths

**Figure 8:**
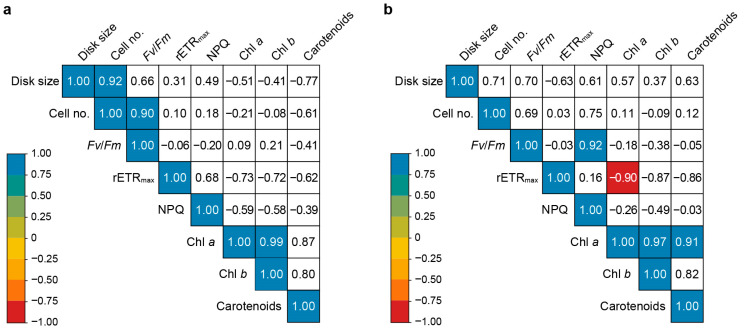
Pearson’s correlation analysis of various parameters in *U. australis* as a function of growth conditions. (**a**,**b**) Pearson’s correlation coefficients for growth rate, photosynthetic efficiency, and photosynthetic pigment contents under different wavelengths (white, blue, green, red, and white light with blue wavelengths filtered out) and (**a**) aerated or (**b**) static conditions. Only indicators showing statistical significance at the 5% level are shown in coloured squares. The colour of the square indicates the strength of the correlation. The coloured bar to the left indicates the scale of the correlation coefficients. The numbers in boxes are the correlation coefficients. Disk size, in mm^2^; cell no., number of cells·2500 μm^−2^; *Fv/Fm*, maximum quantum yield; rETR_max_, relative maximum electron transport rate; NPQ, non-photochemical quenching; Chl *a*, chlorophyll *a* content (mg·mgFW^−1^); Chl *b*, chlorophyll *b* content (mg·mgFW^−1^); and carotenoids, carotenoid content (mg·mgFW^−1^).

Pearson’s correlation analysis of the growth, photosynthetic efficiency, and photosynthetic pigment content of *U. australis* under various light conditions revealed no relationship between growth (disk size or number of cells) and photosynthetic efficiency (*Fv/Fm*, rETR_max,_ or NPQ) under aerated or static culture conditions (Figure 8).

Under aerated conditions, the disk size exhibited a strong positive correlation with cell number. A strong positive correlation between *Fv/Fm* (a measure of light-harvesting capacity) and NPQ (a mechanism for dissipating excess energy) was detected, whereas rETR_max_ (a measure of electron transport efficiency) was negatively correlated with Chl *a* (a pigment that absorbs light) content.

### 2.5. Relative Efficiency of Blue Light Quanta

**Figure 9:**
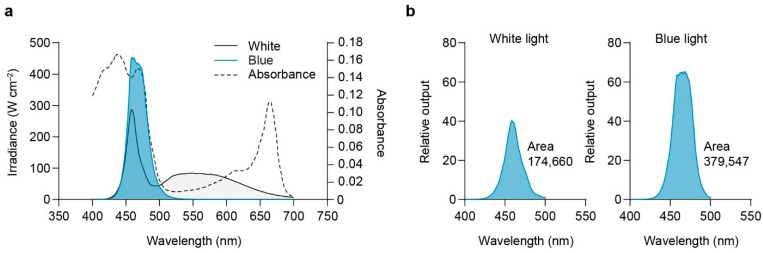
Comparative analysis of white and blue light spectra and blue photon absorption in *U. australis*. (**a**) Spectra of white and blue light and absorption spectral curve. (**b**) Effective number of blue photons (blue-filled) for the white and blue light sources.

The distribution of the spectra emitted from full white and blue light sources is depicted in Figure 9. The effective photon density of the blue light source was 2.17 times greater than that of the white light source.

**Figure 10:**
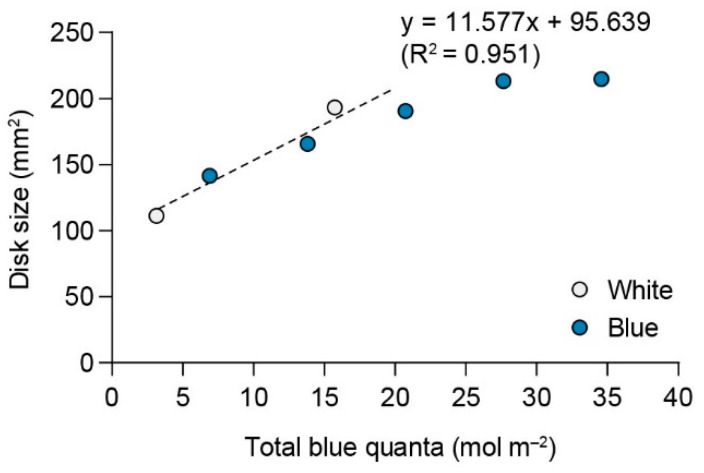
Disk size of *U. australis* as a function of total blue quanta from blue and white light. Cultures were irradiated for 96 h with blue light (20 to 100 μmol photons m^−2^s^−1^) or white light (20 and 100 μmol photons m^−2^s^−1^) over a 12 h light/dark photoperiod. The regression line is expressed by y = 11.557x + 95.639 (*r*^2^ = 0.951).

Figure 10 illustrates the relationship between total blue quanta and disk size cultured under white and blue light of varying intensities. The regression equation fitted to non-saturating values estimated a 50% response at a quantum dose of 1.02 mol m^−2^ for blue light.

### 2.6. Changes in the U. australis Proteome under Static or Turbulent Conditions and Blue Light Illumination

**Figure 11:**
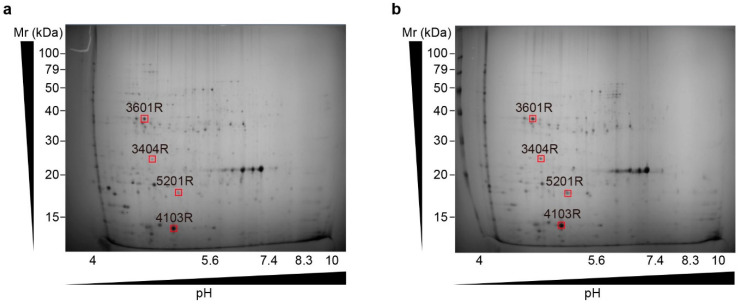
Comparative two-dimensional gel electrophoresis (2DE) of *U. australis* proteins under (**a**) static and (**b**) aerated conditions with blue light illumination. The first dimension comprised a 24 cm nonlinear immobilised pH gradient (IPG) of pH 4–10 and isoelectric focusing. The second dimension comprised a 20 × 24 cm 10–16% sodium dodecyl sulphate–polyacrylamide gel electrophoresis (SDS-PAGE). Proteins were detected by Coomassie Brilliant Blue staining. The nonlinear pH range of the first-dimension IPG strip is indicated at the bottom of the gel, with acidic pH values on the left. The relative molecular mass (Mr scale) was used to estimate the molecular weights of the separated proteins. The red squares indicate protein spots with higher abundance under aerated conditions compared with static conditions.

The proteomic changes underlying the various growth kinetics observed during various culture conditions were evaluated by performing a proteomic analysis of differentially abundant proteins after two-dimensional sodium dodecyl sulphate–polyacrylamide gel electrophoresis (SDS-PAGE). Four of the ninety-four protein spots increased in abundance under blue light and aerobic culture conditions (Figure 11, Table 1).

**Figure 12:**
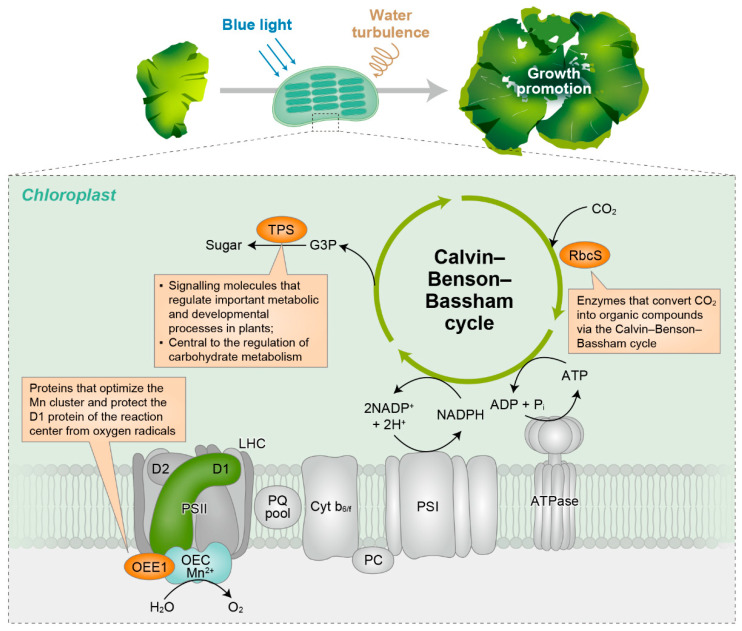
Simplified model of protein accumulation in the photosynthetic electron transport chain and Calvin–Benson–Bassham cycle in *U. australis* under interacting culture conditions of blue light and water turbulence.

Matrix-assisted laser desorption/ionisation time-of-flight (MALDI-TOF)/TOF analysis revealed a ribosomal housekeeping protein localised within the chloroplast (spot no. 3601). In addition, three other proteins (spot nos. 3404, 4103, and 5201) that function in photosynthetic metabolism were identified (Figure 12). Spots 3404, 4103, and 5201 correspond to extrinsic oxygen-evolving enhancer protein 1 (OEE1), ribulose biphosphate carboxylase small subunit (RbcS), and trehalose-6-phosphate synthase (TPS), respectively.

## 3. Discussion

### 3.1. Growth and Cell Number

Significant differences were observed in the growth of *U. australis* disks exposed to various light wavelengths under aerated conditions. Both white and blue light yielded higher growth rates than green and red light. Notably, under static conditions, there were no significant differences in growth rates among the various light treatments. These results highlight the significant role of aeration and turbulence in conjunction with specific light wavelengths for promoting *U. australis* growth.

The role of blue light was further investigated by culturing *U. australis* under white light with or without the blue waveband. We found that the growth of *U. australis* disks was considerably reduced when cultured under white light devoid of its blue waveband compared with when grown under full white light. The smaller size of the disks in the absence of the blue waveband provides compelling evidence of the significant influence of blue light in promoting *U. australis* growth. These findings emphasise the interactive effects of blue light and water turbulence in fostering *U. australis* growth.

Previous studies have reported similar growth responses to blue light among different *Ulva* spp. Le et al. [49] demonstrated that *U. australis* exhibited superior growth under aerated conditions when exposed to blue light than upon exposure to red light. Gong et al. [7] reported higher growth rates in *U. lactuca* under aerated culture conditions with blue-light-emitting diode (LED) illumination than those under fluorescent light. These studies, in conjunction with our findings, highlight the positive effect of blue light on the growth of *Ulva* spp.

The cell density evaluation revealed intriguing patterns in response to different light conditions. Under aerated conditions, white and blue light yielded significantly higher cell densities compared with green and red light. Conversely, under static conditions, there were no significant differences in the number of *U. australis* cells cultured under different wavelengths of light. The absence of the blue waveband resulted in a lower cell density than that under full white light under both aerated and static conditions.

Thallus growth in *U. australis* is intricately influenced by the interplay between cell division and growth [50]. Our observations suggest that the larger disk sizes observed under white and blue light with aeration may have primarily resulted from active cell division rather than cell elongation. Although the existing reports on the effects of blue light on cell division in algae are inconsistent, the findings from this study support the role of blue light in stimulating cell division in *Ulva* spp. Kuwano et al. [51] demonstrated that blue light illumination provided the most effective conditions for opening the G1 gate during cell division in *U. compressa*. Our findings on *U. australis* align with the aforementioned observations, offering additional evidence of the involvement of blue light in stimulating cell division in *Ulva* spp. However, the related molecular mechanisms require further investigation.

### 3.2. Photosynthetic Pigment Contents

Pigment accumulation patterns in *U. australis* exhibited intriguing characteristics under various light conditions. The lowest pigment concentrations were observed under blue light exposure. The photosynthetic pigment contents were comparable under aerated and static culture conditions for both full white light and white light devoid of the blue component. These results correspond with the behaviour of terrestrial plants under blue light, exhibiting sun-type traits, a lower chlorophyll content, and reduced thylakoid structures [52]. Therefore, blue light in conjunction with aeration may elicit a sun-type response in *U. australis*, potentially representing an adaptive energy-saving strategy for the high light intensity conditions in the intertidal zone, where *U. australis* thrives.

Notably, *U. australis* exhibited a remarkable capacity to accumulate pigments under green light as opposed to other light wavelengths, although green algae exhibit poor light absorption and photosynthesis efficiency in this particular range of the light spectrum compared with under other spectra [53]. It is noteworthy that even green LEDs contain some blue wavelengths, and this inclusion of blue wavelengths in green light may have unexpected effects on pigment levels. This phenomenon could potentially be associated with a green-light-induced shade-avoidance response, akin to that observed in terrestrial plants. Notably, unshaded plants exposed to additional green light exhibit growth patterns similar to plants grown in shade. *U. australis* probably perceives this green light signal and responds by enhancing pigment production, thereby optimising light capture for photosynthesis. In line with these observations, an increase in thylakoid structure and photosynthetic pigment contents in response to blue-green light has been reported in other green algae and marine microalgae [54,55]. This green light response is mediated by a signalling pathway that does not rely on currently known photoreceptors [56].

Furthermore, chlorophyll levels in *U. australis* were increased under red light, consistent with previous research findings on *U. rigida*, where red- and far-red-light-absorbing photoreceptors, such as phytochromes, were implicated in the induction of chlorophyll biosynthesis [57].

Green light considerably stimulated carotenoid biosynthesis in *U. australis*. Carotenoids play vital roles in light harvesting and protection against photooxidative damage in photosynthetic organisms [58,59]. Carotenoid production is precisely regulated by various signals, including light [60,61]. The elevated carotenoid levels in *U. australis* exposed to green light in the current study may be attributed to increased chlorophyll production as part of a shade-like response. The simultaneous increase in carotenoid and chlorophyll levels under green light exposure suggests an adaptive response aimed at enhancing light capture under perceived shade conditions.

Previous studies on microalgae have reported similar light-mediated regulation of carotenoid biosynthesis and wavelength-dependent translation of carotenoid-related proteins. For instance, blue light promotes the accumulation of specific carotenoids in certain green algal species [59]. However, the underlying mechanisms at the transcription factor level and the species–specific effects of specific light spectra on carotenoids remain poorly understood and present exciting avenues for future research.

### 3.3. Chl a Fluorescence

Efficient photosynthesis is essential for the growth and survival of photosynthetic organisms. Our experiments revealed interesting patterns in the photosynthetic performance of *U. australis* under various light conditions. Higher *Fv/Fm* values were observed in the aerated cultures under white and blue light than under green and red light. This is consistent with previous findings demonstrating a higher quantum yield of photosystem II (PSII) under blue light in various photosynthetic organisms [17,62,63]. The enhanced *Fv/Fm* values observed in *U. australis* may be attributed to the efficient absorption of blue light by PSII. Moreover, the significant variation in *Fv/Fm* values between light wavelengths under static conditions suggests that *U. australis* has dynamic regulatory mechanisms to optimise its photosynthetic efficiency based on the available light resources.

*U. australis* displayed significantly higher photosynthetic capacity (rETR_max_) and light-harvesting efficiency (*Fv/Fm*) under blue light than under other wavelengths. This suggests that under blue light conditions, *U. australis* has evolved unique adaptations and an optimal balance of PSII and PSI. Individual Chl *a* molecules with low mutual shading may contribute to the improved light-harvesting capacity and overall photosynthetic performance observed under blue light [62]. These findings emphasise that efficient utilisation of blue light for electron transport ultimately results in higher rates of photosynthesis and growth in *U. australis*.

NPQ serves as a vital photoprotective strategy to dissipate excess photon energy as heat and prevent photooxidative damage to chloroplasts. Irrespective of aeration, *U. australis* exhibited higher NPQ levels under blue light than under other wavelengths in the present study. The increased light harvesting and subsequent electron transport associated with blue light exposure may have resulted in hydrogen atom accumulation in the thylakoid lumen, a steep pH gradient between the chloroplast stroma and the lumen, and the dissipation of excess energy as heat. The higher NPQ levels in *U. australis* suggest that blue-light-induced heat dissipation possibly serves as an important regulatory mechanism to maintain effective photosynthetic performance even under excessive light.

### 3.4. Correlation between the Growth, Photosynthetic Efficiency, and Photosynthetic Pigment Contents of U. australis under Different Light Wavelengths

In both the aerated and static cultures, there was no significant correlation between growth parameters (disk size or cell number) and photosynthetic efficiency (*Fv/Fm*, rETR_max_, or NPQ). This suggests that factors beyond photosynthesis influence the growth of *U. australis.* Understanding the intricate relationship between growth, photosynthetic efficiency, and photosynthetic pigment content is fundamental for unravelling the mechanisms that drive the productivity and performance of photosynthetic organisms. Lüning [64] emphasised that the multifaceted nature of algal growth is simultaneously determined by nutrient availability, CO_2_ levels, and light-dependent non-photosynthetic processes. Nevertheless, the unanticipated results obtained in our study indicate that the observed growth stimulation induced by blue light in *U. australis* may involve additional morphogenetic responses.

Under aerated conditions, a strong positive correlation between disk size and cell number was observed, indicating that the larger disk size observed under the combined influence of blue light and water turbulence was primarily a consequence of increased cell division. These findings elucidate the pivotal role played by blue light and water turbulence in promoting cell proliferation and overall growth in *U. australis.*

The strong positive correlation between *Fv/Fm* (a measure of light-harvesting capacity) and NPQ (a mechanism for dissipating excess energy) observed in this study suggests that greater energy transfer to the reaction centre activates the xanthophyll cycle, thereby enhancing photoprotection. Thus, the positive correlation between *Fv/Fm* and NPQ underscores the concerted regulation of these processes to optimise light utilisation and safeguard against photooxidative damage.

Additionally, a negative correlation was observed between rETR_max_ and the Chl *a* level, which suggests that the excessive accumulation of light-harvesting pigments, such as Chl *a*, may exacerbate self-shading effects, ultimately diminishing the electron transport efficiency. Notably, this effect was most pronounced in *U. australis* cultivated under green light, where the highest chlorophyll concentrations paired with the lowest rETR_max_ values were observed. These results emphasise the importance of maintaining an optimal balance between light absorption and electron transport efficiency for maximising photosynthetic performance in *U. australis*.

### 3.5. Relative Efficiency of Blue Light

A substantial difference in the spectral distribution of light emitted from full white and blue light sources was observed, indicating that the blue light source has a considerably higher quantum number than the full white light source even when subjected to equivalent photon irradiance. This highlights the remarkable efficiency of blue light in delivering photons to *U. australis*.

The correlation analysis between total blue quanta and disk size under varying intensities of white and blue light revealed that a blue quantum dose of 1.02 mol m^−2^ elicited a 50% growth response. Similar blue light responses have been previously observed in *U. australis* [18] as well as in brown algae [12,15,65,66,67,68].

Although a detailed investigation of the action spectrum for the response to blue light was performed, the observed quantum requirements for a 50% growth response aligned with previous findings (Table 2), further supporting the potential involvement of cryptochrome in *U. australis* growth. Cryptochromes are ubiquitous in brown algae and *U. australis*, where they mediate blue light responses [18,69]. In *U. australis*, a cryptochrome has been proposed to participate in the reactivation of spore germination following UV-B exposure [18].

### 3.6. Changes in the U. australis Proteome with or without Aeration under Blue Light

The upregulated expression of a housekeeping protein (spot no. 3601) indicated that the observed changes in protein abundance were not solely limited to photosynthetic metabolism. Additionally, OEE1, RbcS, and the thermal processing unit (TPS), which are intrinsic to photosynthetic processes, were detected.

The protection of the D1 protein from oxygen radical damage and the optimisation of Mn cluster function during photosynthetic oxygen evolution are both dependent on OEE1 [70]. Photosynthesis and photorespiration are significantly influenced by ribulose-1,5-bisphosphate carboxylase/oxygenase (RuBisCO), which is essential for CO_2_ conversion to organic compounds [71]. TPS modulates plant metabolism and growth through sugar signalling [72], and elevated TPS levels promote growth and gene expression [72,73]. TPS also regulates starch metabolism and end-product accumulation in terrestrial plants [74,75,76].

Thus, the identification of these proteins related to photosynthetic metabolism and sugar signalling indicates that *U. australis* has evolved distinct mechanisms to optimise photosynthetic efficiency and coordinate metabolic processes for enhanced growth under blue light and aerated conditions. Nevertheless, further investigations are needed to unravel the precise roles of these proteins and their interactions in *U. australis*.

## 4. Materials and Methods

### 4.1. Laboratory Culture of U. australis

*U. australis* samples were collected from the Gijang-gun region, Busan, on the southern coast of the Republic of Korea (35°17′59″ N, 129°15′35″ E). Samples were transported to the laboratory and immediately stored at 15 °C in artificial seawater in a 250 L aquarium equipped with an air pump.

Prior to the experiment, at least seven adult *U. australis* thalli of similar sizes (total length of approximately 15 cm) were selected. Algal disks (Ø 12 mm) were cut from the centre of each *U. australis* thallus and transferred to a 500 mL glass container filled with 450 mL of artificial seawater [41]. The algal disks were stored at 15 ± 1 °C under 20–30 μmol photons m^–2^s^–1^ of fluorescent light (FL20SS; Philips, Eindhoven, The Netherlands) with a 12 h light/dark photoperiod for 24 h under aerated conditions prior to light treatment.

### 4.2. Experimental Treatments

**Figure 13:**
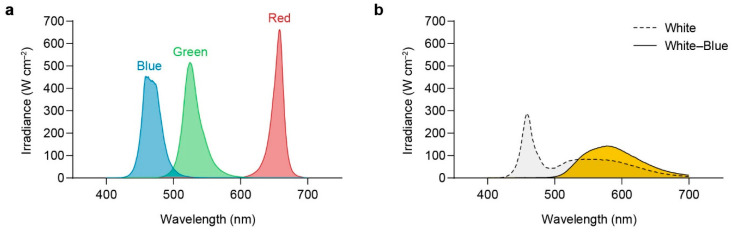
Emission spectra of light sources used in this study. (**a**) Blue, green, and red light wavelengths. (**b**) White light and white light depleted of the blue wavelength component using a broadband filter.

LED panels (with white, blue, green, or red LEDs; 340 × 500 × 10 mm; Daewon, Bucheon, Republic of Korea) were used as light sources. The emission spectrum of each light source was measured using a spectroradiometer (Avaspec-ULS2048; Avantes, Apeldoorn, Gelderland, The Netherlands). Figure 13 depicts the emission spectra for white (400–700 nm), blue (emission peak at 460 nm), green (emission peak at 525 nm), and red light (emission peak at 660 nm) and the emission spectrum for white light depleted of blue light, which was accomplished using a broadband filter (015; LEE Filters, Andover, UK). Photon irradiance was measured using a quantum sensor (LI-1400; LI-COR, Lincoln, NE, USA) to ensure that all light treatments had the same irradiance (100–110 μmol photons m^−2^s^−1^).

For the experimental treatments, 12 *U. australis* disks were placed in 250 mL Erlenmeyer flasks (*n* = 3 replicates per condition), each containing 200 mL of Ott’s medium [77], for aerated and static cultures. The flasks were illuminated with white, blue, green, or red light at 100–110 μmol photons m^–2^s^–1^ under a 12 h light/dark photoperiod for 4 days, with the culture temperature maintained at 15 ± 1 °C. The water turbulence produced by aeration caused gentle algal movement, and the Ott’s medium was changed every 2 days. After 4 days, the disks were harvested for morphological, photosynthetic, and biochemical evaluation.

### 4.3. Measuring Growth and Cell Number

The growth of the *U. australis* disks was measured using an image analyser (Moticam 2.0; Ted Pella, Redding, CA, USA). The number of cells in a given area (2500 μm^2^) was counted by examining the surface view of *U. australis* disks under a microscope (Axioskop 2 Plus; Zeiss, Jena, Germany).

### 4.4. Estimating Pigment Content

The photosynthetic pigment contents of the *U. australis* disks were determined using a spectrophotometer (S-3100 PDA UV-Vis; Scinco, Seoul, Republic of Korea). Pigments were extracted from the disks in 1 mL methanol (≥99.9%; CAS No. 67-56-1; Sigma-Aldrich, St. Louis, MO, USA) for 24 h at 4 °C in the dark. The absorbance values of the methanolic extracts were measured at 666, 653, and 470 nm using a spectrophotometer to estimate the Chl *a*, Chl *b*, and carotenoid pigments, and Equations (1)–(3), as described by Lichtenthaler [78], were used for calculating their contents:

Chl *a* content = 15.65 × A_666_ − 7.34 × A_653_ (1)

Chl *b* content = 27.05 × A_653_ − 11.21 × A_666_; (2)

Carotenoid content = (1000A_470_ − 2.86 × Chl *a* − 129.2 × Chl *b*)/245 (3)

where A represents the absorbance values at the respective wavelengths. In Equation (3), Chl *a* and Chl *b* represent the respective pigment contents obtained using Equations (1) and (2).

### 4.5. Measuring Chl a Fluorescence

Chl *a* fluorescence, an indicator of light-dependent photosynthetic responses in PSII [18], was measured using a pulse-amplitude-modulation imaging fluorometer (Walz, Effeltrich, Germany). After 4 days of growth under the experimental treatments described previously, the *U. australis* disks were placed in 24-multi-well plates (30024, SPL, Pocheon, Republic of Korea) and dark-adapted for 15 min. The samples were then irradiated with LED pulses (0.15 µmol photons m^−2^s^−1^) to determine the initial fluorescence yield (*Fo*). The maximum fluorescence yield (*Fm*) was determined by irradiating with a saturating pulse of approximately 5000 µmol photons m^−2^s^−1^ emitted by a built-in halogen lamp. The maximum photosynthetic quantum yield (*Fv/Fm*) was calculated as (*Fm* − *Fo*)/*Fm*.

The electron transport rates were determined from rapid light curves, which provide an estimate of the saturation properties of electron transport and the overall photosynthetic performance of photosynthetic organisms [79]. Curves were derived using actinic light pulses of 10 s duration with a stepwise increase from 0 to 531 µmol photons m^−2^s^−1^. The hyperbolic tangent equation shown in Equation (4) was used to determine rETR_max_, as described by Platt et al. [80]:

rETR_max_ = (1 − exp[−α × I/P_t_]) × exp(−β × I/P_t_), (4)

where α denotes the electron transfer rate under light-limited conditions, P_t_ represents a theoretical parameter, and β is the inhibition coefficient.

NPQ was estimated as NPQ = (*Fm* − *F′m*)/*F′m* [81], where *F′m* is the maximum fluorescence yield under light-adapted conditions.

### 4.6. Relative Effectiveness of the Blue Quantum

To compare the relative effectiveness of the blue quanta emitted by white and blue light, the spectral energy output from 400 to 700 nm (white) and 400 to 500 nm (blue) was converted into quanta [65]. The quanta of 1 nm wavelength bands were expressed as a ratio relative to the total sum. The resulting curves were multiplied by the absorbance spectral curves of *U. australis* at the corresponding wavelengths, and the areas under the curves were subsequently compared.

### 4.7. Protein Extraction from U. australis Disks

Twelve *U. australis* disks grown under turbulent or static conditions in the presence of blue light were harvested and homogenised in liquid nitrogen using a motor-driven homogeniser (PowerGen 125, Fisher Scientific, Hampton, NH, USA). Homogenates were lysed in 10 volumes of lysis solution consisting of 7 M urea, 2 M thiourea, 4% (*w*/*v*) 3-[(3-cholamidopropyl) dimethylammonio]-1-propane sulfonate (CHAPS), 1% (*w*/*v*) dithiothreitol (DTT), 2% (*v*/*v*) pharmalyte, and 1 mM benzamidine. Proteins were extracted by shaking the lysate for 1 h at 25 °C, followed by centrifugation at 15,616× *g* for 1 h at 25 °C. The supernatant was subjected to two-dimensional electrophoresis, and the protein concentration was quantified using the Bradford colorimetric assay [82].

### 4.8. Two-Dimensional Electrophoresis and Image Acquisition

Protein extracts (400 μg) were separated in the first dimension by isoelectric focusing using an immobilised pH gradient dry strip (24 cm, pH 4–10, Genomine DryStrip; Genomine, Pohang, Republic of Korea) and equilibrated with a rehydration solution (comprising 7 M urea, 2 M thiourea, 2% (*w*/*v*) CHAPS, 1% (*w*/*v*) DTT, and 1% (*v*/*v*) pharmalyte) for 12–16 h, followed by 2D SDS-PAGE (20 × 24 cm). Proteins were detected using Coomassie Brilliant Blue staining. Image analysis and quantification of protein spots were performed using PDQuest software version 8.0 (Bio-Rad Laboratories, Hercules, CA, USA). The amount of protein in each spot was normalised to the total intensity of valid spots.

### 4.9. Protein Identification by MALDI-TOF/TOF

For protein identification and peptide mass fingerprinting (PMF), protein spots were excised and digested with trypsin (Promega, Madison, WI, USA), mixed with α-cyano-4-hydroxycinnamic acid in 50% (*v*/*v*) acetonitrile + 0.1% (*v*/*v*) trifluoroacetic acid, and subjected to MALDI-TOF/TOF (Autoflex maX; Bruker, Bremen, Germany) with LIFT^™^ ion optics. The mass list of PMF was analysed using Mascot (version 2.1, Matrix Science, London, UK) to search for matching proteins in the National Centre for Biotechnology Information Non-Redundant database using the following parameters: trypsin as the cleaving enzyme, maximum failed cleavage, iodoacetamide as the full modification of cysteine, oxidation of methionine as the partial modification, monoisotopic masses, and mass tolerance of 0.1–0.2 Da.

### 4.10. Statistical Analysis

Differences between treatments were determined using ordinary two-way analysis of variance with a randomised complete block design (no repeated measures), followed by a post hoc least significant difference test. Results are presented as mean with 95% confidence intervals (shown as error bars where relevant). Correlations between growth and physiological parameters were assessed using Pearson’s correlation coefficient calculated with the ggplot2 package [83] in R (version 4.0.5; R Foundation for Statistical Computing, Vienna, Austria). For all tests, *p* < 0.05 was considered statistically significant.

## 5. Conclusions

In summary, the results of this study indicate that blue-light-induced growth enhancement in the presence of water turbulence resulted from active cell division rather than photosynthetic efficiency, implying that blue-light-mediated growth promotion may be a photomorphogenic response likely involving the blue light photoreceptor cryptochromes. In particular, three proteins related to photosynthetic metabolism and sugar signalling were activated.

The growth-promoting effect of blue light on *U. australis* in the presence of water turbulence can be attributed to the absorption of blue light quanta (e.g., 1.02 mol m^−2^) by cryptochromes. This light-induced molecular response may lead to developmental changes, such as growth, which improves the ability of the alga to respond to water turbulence. The precise mechanism of this cryptochrome signal transduction pathway and its effects on water turbulence sensitivity remain unknown and require further investigation. Furthermore, several proteins in *U. australis* that accumulate upon exposure to a combination of blue light and water turbulence were identified. These proteins were related to chloroplast function, implying that more photosynthesis-related proteins become available for plastid differentiation under these culture conditions, potentially facilitating efficient photosynthesis and contributing to the enhanced growth of *U. australis*.

These insights have important practical implications for optimising the growth and productivity of this valuable marine organism. Investigating the nature of the blue light effect, unravelling the underlying molecular mechanisms, and assessing the broader applicability of these findings will advance our understanding of *Ulva* cultivation and contribute to the sustainable growth of the aquaculture industry. Future studies should consider the use of non-targeted metabolomics to characterise metabolite accumulation differences and elucidate the regulatory mechanisms underlying the growth-promoting effects of blue light and water turbulence in *U. australis*.

## Figures and Tables

**Table 1:** Protein spots identified by matrix-assisted laser desorption/ionisation (MALDI)-time-of-flight (TOF)/TOF analysis of *Ulva australis* disks grown under blue light and aerated conditions.

Spot Number	Molecular Weight (kDa)	Protein Name	Gene Symbol	Description
3601	37.2	30S ribosomal protein S9, chloroplast	*rps9*	Housekeeping gene/reference gene
4103	13.4	Ribulose bisphosphate carboxylase small subunit	*RbcS*	An enzyme that converts CO_2_ into organic compounds via the Calvin–Benson–Bassham cycle
5201	17.5	Trehalose-6-phosphate synthase	*TPS*	Synthesises a signalling molecule that regulates plant metabolism and development; central to the regulation of carbohydrate metabolism
3404	24.2	Oxygen-evolving enhancer protein (Fragment)	*OEE1*	Optimises the Mn cluster and protects the D1 protein of the reaction centre from oxygen radicals

**Table 2:** Quantum requirements for a 50% response of photomorphogenic measures in marine algae.

Species	Quantum Requirement (mol·m^−2^)	Response	Reference
*Alaria esculenta*	1.9	Photoreactivation	Han and Kain [12]
*Laminaria hyperborea*	2.5	Photoreactivation	Han and Kain [65]
	2.1	Egg formation	Dring and Lüning [66]
*Laminaria saccharina*	1.95	Egg release	Lüning and Dring [15]
	1.2	Photoreactivation	Han and Kain [12]
*Macrocystis pyrifera*	2.6	Egg formation	Lüning and Neushul [67]
*Scytosiphon lomentaria*	2.25	2D ^1^ growth	Dring and Lüning [68]
	1.97	Hair formation	Dring and Lüning [68]
*Ulva australis*	1.02	Growth	This study

^1^ 2D, two-dimensional.

## Data Availability

The data presented in this study are available on request from the corresponding author. The data are not publicly available.
